# Antifungal, antitoxigenic, and antioxidant activities of the essential oil from laurel (*Laurus nobilis* L.): Potential use as wheat preservative

**DOI:** 10.1002/fsn3.1650

**Published:** 2020-07-23

**Authors:** Azem Belasli, Yamina Ben Miri, Malek Aboudaou, Lidia Aït Ouahioune, Luis Montañes, Agustín Ariño, Djamel Djenane

**Affiliations:** ^1^ Laboratoire de Qualité et Sécurité des Aliments Département Technologie Alimentaire Université Mouloud MAMMERI de Tizi‐Ouzou Tizi‐Ouzou Algeria; ^2^ Département Recherche & Développement ISO 9 International Isser Algeria; ^3^ Valero Analítica Zaragoza Spain; ^4^ Facultad de Veterinaria Instituto Agroalimentario de Aragón—IA2 (Universidad de Zaragoza‐CITA) Zaragoza Spain

**Keywords:** aflatoxin B1, antifungal activity, antioxidant activity, *Aspergillus flavus*, essential oil, laurel, wheat grains

## Abstract

Essential oils (EOs) are widely used in the food industry as natural food preservatives to extend product shelf life and as flavoring agents. The aim of this work was to study the chemical profile of the EO from laurel (*Laurus nobilis*) and its antifungal, antitoxigenic, and antioxidant activities. The extractive yield of the EO from Algerian laurel was 1.13% being 1,8‐cineole the most dominant compound (35.5%) by gas chromatography–mass spectrometry analysis. The values of minimum inhibitory concentration and minimum fungicidal concentration (MFC) against *Aspergillus flavus* were 1.75 and 2 mg/ml, respectively. The production of aflatoxin B1 was inhibited by EO concentrations between 0.25 mg/ml (15% decrease) and 1.50 mg/ml (86% decrease), and it was totally inhibited at the MFC value. The EO showed a wide antifungal spectrum against other species in a dose‐dependent manner. In a food‐model study, the *L. nobilis* EO showed remarkable efficacy in fumigated wheat grains, providing from 51.5% to 76.7% protection against *A. flavus* during 6‐month storage. The *L. nobilis* EO showed good free radical scavenging activity by DPPH assay (IC_50_ value of 602 μg/ml) and moderate antioxidant activity in the β‐carotene bleaching assay (46% inhibition of linoleic acid oxidation). The conclusions of this study justify future research for the application of EO from laurel as a natural preservative to improve food safety and extend shelf life by controlling spoilage and toxigenic molds as well as oxidative damage.

## Graphical abstract

Briefly, the essential oil (EO) was obtained from leaves of Algerian *Laurus nobilis* (*L. nobilis* EO), and lots of stored wheat (*Triticum aestivum*) grains (moisture content 12%) samples were fumigated with the EO at the minimum inhibitory concentration value. Different lots of stored wheat were inoculated with pathogenic fungi (*Aspegillus flavus*) and maintained for 6 months at 15°C and 62% relative humidity. The *L. nobilis* EO was chemically characterized, and stored wheat samples were analyzed for *Aspegillus flavus* growth and aflatoxin B1 production.

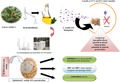

## 1 INTRODUCTION

The aflatoxins are mycotoxins produced primarily by toxigenic strains of the fungi *Aspergillus flavus* and *Aspergillus parasiticus*. The most frequently found aflatoxin in contaminated food samples is aflatoxin B1 (AFB_1_), which is a genotoxic and carcinogenic substance (IARC, 2012). In Africa, about 250,000 hepatocarcinoma‐related deaths occur annually due to aflatoxin ingestion (Wild & Gong, 2010). Aflatoxins can contaminate cereals, oilseeds, nuts, spices, legumes, dried fruits, and milk posing a high health risk (Asi, Iqbal, Ariño, & Hussain, 2012; Iqbal, Asi, Ariño, Akram, & Zuber, 2012; Yassin, El‐Samawaty, Moslem, Bahkali, & Abd‐Elsalam, 2011). Aflatoxin‐producing fungi are found in areas with a hot, humid climate, and aflatoxins in food are a result of both pre‐ and postharvest fungal contamination. The rate and degree of contamination depend on temperature, humidity, soil, and storage conditions (EFSA, 2007). Climate change is anticipated to impact on the presence of aflatoxins in food in Europe.

The Joint FAO/WHO Expert Committee on Food Additives has recently calculated international estimates of chronic dietary exposure to aflatoxins (FAO/WHO, 2018). The mean upper bound (UB) dietary exposure to aflatoxins ranged from 1.3 ng/kg body weight per day (many European countries) to 34.8 ng/kg body weight per day (many African countries). For many European countries, wheat was the main contributor to the UB dietary exposure to aflatoxins, which reached between 37% and 76.5%.

The frequency of contamination of world crops by aflatoxins shows that the strategies currently used are insufficient to guarantee the security of the foods and that it is necessary to develop others, as a complement or substitution of those already existing (Byakika et al., 2019; Lasram et al., 2019; Yassein, El‐Said, & El‐Dawy, 2020). In this context, strategies based on the use of compounds naturally recognized as not harmful to the environment and to health, seem interesting. Indeed, plants produce different secondary metabolites for their protection against pathogenic attacks and environmental stresses (mechanical, biological, or climatic). These compounds could possibly be used as a means of combating fungal contamination and/or mycotoxins (Kedia, Prakash, Mishra, & Dubey, 2014). Essential oils (EOs) are molecules of natural origin, biodegradable and are therefore considered as a possible alternative to synthetic pesticides (Ben Miri, Ariño, & Djenane, 2018). In view of their different biological properties, EOs have shown important antimicrobial activities that can be used for the control of the contamination by molds and mycotoxins in agricultural commodities (Abd El‐Aziz, Mahmoud, Al‐Othman, & Al‐Gahtani, 2015; Ben Miri et al., 2018; Bluma, Amaiden, Daghero, & Etcheverry, 2008; Tian et al., 2012). EOs can also be used as flavoring or food additives (Taoudiat, Djenane, Ferhat, & Spigno, 2018), as well as antioxidants and antibacterial agents in foods (Djenane, Gómez, Yangüela, Roncalés, & Ariño, 2019), whose utilization has been authorized in the USA (FDA, 2019).

*Lauraceae* comprises numerous aromatic and medicinal plants, including *Laurus nobilis*, commonly known as laurel or bay laurel. It is a plant native to the southern Mediterranean region that grows to a height from 6 to 10 m, which is grown commercially for its aromatic leaves. *L*. *nobilis* is one of the cultivated and endemic species to Algeria, which is used mostly in cuisine and traditional medicine. The EO from Algerian wild‐growing laurel has shown effective antimicrobial and antioxidant properties (Boughendjioua, 2017). A direct relationship between the content of bioactive phenols and the biological activity of the *L*. *nobilis* EO has been previously reported, in which 1,8‐cineole was identified as a major phenolic compound (Caputo et al., 2017). However, it is difficult to correlate the antifungal activity of EOs with single compounds. There is evidence that even minor components play a critical role in antimicrobial activities, and it appears that the inhibitory effects are the result of their synergistic action (Cabral, Pinto, & Patriarca, 2013; Djenane et al., 2019).

The aims of the present study were to investigate the antifungal, antitoxigenic, and antioxidant activities of the EO from laurel and to assess the potential use as wheat grain preservative during long storage.

## 2 MATERIALS AND METHODS

### 2.1 Chemicals and equipment

Reagents and solvents chloroform, methanol, dimethyl sulfoxide (DMSO), isoamyl alcohol, hexane, anhydrous sodium sulfate (Na_2_SO_4_), linoleic acid, sodium carbonate (Na_2_CO_3_), and butylated hydroxytoluene (BHT) were purchased from Sigma‐Aldrich. Tween 40, tween‐80, silica gel‐G 60, 2,2‐diphenyl‐1‐picrylhydrazil (DPPH), and β‐carotene were purchased from Sigma‐Aldrich. Culture media DRBC agar (Dichloran, Rose Bengal, Chloramphenicol), PDA (Potato Dextrose Agar), and SMKY liquid media (Sucrose, Magnesium sulfate, Potassium nitrate, Yeast extract) were obtained from Sigma‐Aldrich.

Hydrodistillation apparatus, gas chromatography–mass spectrometry (GC‐MS; Agilent Technologies; model 6,850 and 5,973), centrifugation apparatus (Jouan E76), UV lamp (CN‐6, VILBER LOURMAY), spectrophotometer (6,705 UV/Vis, JENWAY), and a light microscope (Motic: BA210) were used in the present investigation.

### 2.2 Plant material, extraction, and chemical characterization of *L*. *nobilis* EO

The fresh aerial parts of *L. nobilis* (only leaves) were collected during sunny days at the end of June 2015, in the province of Tizi‐Ouzou (Mesghana), 100 km north–east from Algiers (Algeria). The geographic coordinates of collection sites were latitude 36°42′42″N, longitude 4°02′45″E, elevation above sea level 206 m, and 40 km from Mediterranean Sea. The identification was firstly given based on their morphological appearances and according to the flora of Algeria (Quézel & Santa, 1963). A voucher specimen was deposited at the Laboratory of Microbial Systems Biology (voucher number LBSM‐BY15). The collected leaves were dried at ambient temperature in shady and dry conditions for 3 months, then packed, and stored in darkness until the extraction of EO.

**Figure 1:**
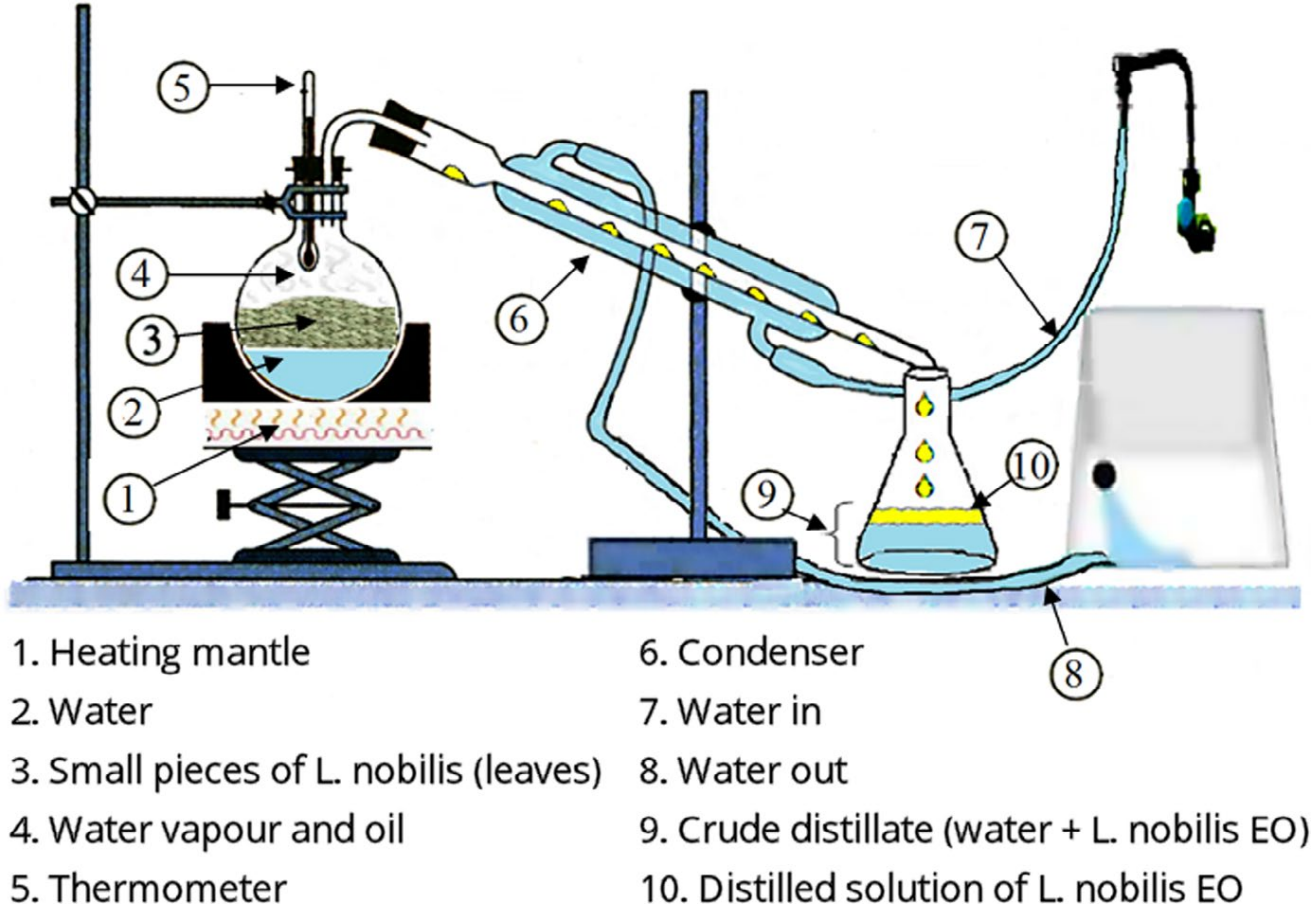
Scheme of *Laurus nobilis* essential oils extraction (hydrodistillation)

Essential oil of two hundred grams (200 g) of dried leaves was extracted by hydrodistillation in Clevenger's apparatus during 3 hr until no more EO was obtained (Figure 1). The water traces in the EO were removed with anhydrous sodium sulfate (Na_2_SO_4;_ Sigma‐Aldrich). EOs were weighted and conserved at 4°C until use. Total phenolic compounds in the *L. nobilis* EO were estimated spectrophotometrically according to the Folin–Ciocalteu method with some modifications (Djenane et al., 2019). Gallic acid (GA) was used as phenolic compound standard for the calibration curve. Results were expressed as micrograms of GA equivalents per milligram of sample dry weight (μg GAE/mg), using the equation of the linear regression line of the calibration curve. The same EO was characterized by GC‐MS (Agilent model 6,850 and 7,890). The EO (10 μl) was dissolved in hexane (100 μl), and 2 μl of the solution was injected on apolar capillary column DB‐5 (length 30 m × 0.25 mm i.d., film thickness 0.25 μm). Helium was used as the carrier gas at a flow rate of 1.0 ml/min. The column inlet pressure was 8.07 psi. The GC column oven temperature was increased from 60 to 245°C at 3°C/min, with a final hold time of 4 min. The operating parameters for the electron ionization–MS were as follows: electron energy, 70 eV; automatic scanning of the mass range 50–550 amu; ion source temperature, 230°C; and quadrupole, 150°C. The identification of volatile constituents was given by comparing their linear retention index according to Van den Dool and Kratz (1963) based on *n*‐alkane indices (C8–C27), with reference to Adams (2007).

### 2.3 Fungal material

*Aspergillus flavus* E73 strain was obtained from the culture collection of the Laboratory of Microbial Systems Biology at Kouba. Additionally, fungal strains of *Aspergillus carbonarius*, *A*. *fumigatus*, *A*. *niger*, *A*. *ochraceus*, *A*. *tamarii*, *A*. *terreus*, *Fusarium* sp., *Penicillium* sp., and *Rhizopus* sp., isolated in our laboratory during mycological analysis of cereals and spices, were used to study the antifungal spectrum of the EO. Spore inoculum of each fungi was prepared from a culture on PDA at 25ºC for 7 days where spores were obtained by washing the Petri dish with 20 ml of 0.1% Tween 80 solution. The number of spores, determined using a hemocytometer slide (depth 0.2 mm, 1/400 mm^2^) under a light microscope, was adjusted to 1 × 10^6^/ml throughout the study.

For the confirmation of the aflatoxinogenicity, 10 μl of spore suspension of *A*. *flavus* E73 strain was cultivated in Erlenmeyer flask containing 25 ml of the SMKY liquid medium at 25ºC for 10 days. The content was filtered (Whatman N° 1) and extracted with 20 ml chloroform. After stirring and then decanting, the chloroform phase was evaporated to 1 ml. A volume of 50 µl was spotted on a thin layer chromatography plate (TLC). The development was carried out in a standard tank (20 × 20 cm) previously saturated with the solvent system toluene‐isoamyl alcohol‐methanol (90/32/2; *v*/*v*/*v*). After migration, the plate was removed and dried at 60°C for 24 hr. AFB_1_ was detected by placing the plate in UV transilluminator (360 nm) in which AFB_1_ appeared as a blue spot. The intensity of the fluorescence of the spots confirmed the presence of AFB_1_ (Mishra et al., 2013).

### 2.4 In vitro antifungal and antiaflatoxigenic assays of *L*. *nobilis* EO

For the antifungal activity of *L*. *nobilis* EO against *A*. *flavus*, different concentrations of EO were added to 15 ml PDA at 45–50°C to obtain final concentrations in the range 0.25–2.00 mg/ml and poured into Petri dishes. Thereafter, 10 μl of spore suspension was spotted in the center of each Petri dish and incubated at 25°C for 7 days. The controls were prepared in parallel without EO. Measurements were made daily by taking the average of two perpendicular diameters of each colony. The comparison of the dimensions obtained with those of the controls made it possible to calculate the percentage inhibition (*I*%) at day 7, according to the following formula:

$$\text{Percentage}\;\text{mycelial}\;\text{inhibition}\;\left(I\%\right)=\frac{\left(C-T\right)\times 100}{C},$$

*C*: average colony diameter (mm) in the control; *T*: average colony diameter (mm) in the treatment.

Additionally, the minimum inhibitory concentration (MIC) and *minimum* fungicidal concentration (MFC) were assessed for a series of foodborne fungi by the broth dilution method (Shukla, Kumar, Singh, & Dubey, 2009). Different concentrations (0.25–2.00 mg/ml) of *L*. *nobilis* EO were added to 10 ml SMKY broth medium in test tubes. Tubes with only SMKY medium (10 ml) were used as controls. The tubes were inoculated with 10 µl of spore suspensions and incubated at 25°C for 7 days. The MIC was determined as the lowest concentration of EO that inhibited fungal growth. After determining the MIC, 100 μl from the corresponding tubes was subcultured on PDA plates for the determination of the MFC. After 72 hr incubation at 25ºC, MFC was determined as the lowest EO concentration that prevented visible growth.

For the antiaflatoxigenic assay, 10 μl of spore suspension of *A*. *flavus* was inoculated in Erlenmeyer flask containing 25 ml of SMKY medium with different concentrations (0.25–2.00 mg/ml) of *L*. *nobilis* EO and incubated at 25°C for 10 days (Mishra et al., 2013) together with the corresponding control tubes without EO. After incubation, the culture media was filtered (Whatman N° 1), and then, the mycelium dry weight was determined after desiccation at 80°C for 12 hr. For the extraction of AFB_1_, the same procedure was followed as described in section of test confirming the aflatoxinogenicity of the strain. The intensity of the fluorescence of the spots confirmed the presence of AFB_1_. For the quantification of AFB_1_, the blue spots on TLC plates were scraped out, dissolved in 5 ml cold methanol, and centrifuged at 3,000 g for 5 min. The absorbance of the supernatant was made using a UV‐visible spectrophotometer at 360 nm. The quantity of AFB_1_ was calculated according to the formula by Tian et al. (2011):

$${\text{AFB}}_{1}\;\text{content}\left(\mu g/\text{ml}\right)=\frac{\left(D\times M\right)}{\left(E\times l\right)}\times 1,000,$$

*D*: absorbance; *M*: molecular weight of aflatoxin (312); *E*: molar extinction coefficient (21,800); *l*: path length (1 cm).

### 2.5 Activity of *L*. *nobilis* EO against *A*. *flavus* growth in stored wheat grains

Two varieties of wheat (*Triticum aestivum)*, namely AS 81,189 A (Ain Abid) and HD 1,220 (Hiddab), harvested during 2016 in Dar El Beida, Algiers (Algeria), were used. The geographic coordinates of collection sites were latitude 36°42′51″ (N), longitude 3°12′45″ (E), elevation above sea level 17 m, and 10 km from Mediterranean Sea. Wheat grains were collected (around 10 kg of each variety), placed in sterile bags, and brought to the laboratory. The moisture content of the grains was 12% as determined by oven drying. The effect of *L*. *nobilis* EO on wheat was evaluated according to the method recommended by Prakash, Singh, Kedia, and Dubey (2012). The wheat grains of each variety were surface‐sterilized with a 1% solution of sodium hypochlorite and rinsed 3–5 times with sterilized distilled water. One kilogram of each variety was kept separately in plastic boxes with an aerial volume of 2 L. One milliliter (1 ml) of spore suspension of *A*. *flavus* was inoculated into grain samples through uniform spraying. Then, the grain samples in the plastic boxes were fumigated with the EO at the MIC value with respect to aerial volume of container. Control samples were prepared in parallel without EO addition. All the containers were kept airtight and stored for 6 months at 15°C and 62% relative humidity.

For mycological analysis, the spread‐plate method was used. Ten grams of treated and control grain samples was placed in 250‐ml flasks containing 90 ml of sterile Tween water (0.1%) and homogenized in Stomacher for 2 min. Serial decimal dilutions up to 10^–3^ were made, and 0.1 ml of each dilution was inoculated on prepoured, solidified DRBC agar plates, and the inoculum spread with a sterile Drigalski spatula. Plates were incubated at 25ºC for 5 days, and the count was expressed as cfu/g of *A*. *flavus*.

### 2.6 Antioxidant activity of* L. nobilis* EO

The antioxidant activity of EO was evaluated by two methods: the DPPH free radical scavenging assay and the β‐carotene/linoleic acid bleaching assay**.** DPPH test evaluates the capacity of the EO to scavenge 2,2‐diphenyl‐1‐picrylhydrazil radical (DPPH•). Briefly, in clean and dry tubes, volumes of 50 µl of different concentrations (100, 200, 400, 600, 800, and 1,000 µg/ml) of *L*. *nobilis* EO and standard BHT were added to 5 ml of 0.004% (w/v) methanolic solution of DPPH and incubated in darkness at room temperature for 30 min. Thereafter, the absorbance was measured against a blank at 517 nm. DPPH radical scavenging activity was expressed in terms of inhibition percentage (*I*%) and was calculated using the following formula:

$$I\%=\left(\frac{A_{\text{blank}}-A_{\text{sample}}}{A_{\text{blank}}}\right)\times 100,$$

*A*_blank_: absorbance of the control; *A*_sample_: absorbance of the sample.

The value of the inhibitory concentration (IC_50_) represents the dose of the EO that causes the neutralization of 50% of the DPPH radicals. IC_50_ was estimated by extrapolation by plotting the percent inhibition (*I*%) versus concentration curves.

The β‐carotene/linoleic acid bleaching assay is a complementary method to assess the antioxidant activity of compounds (Miraliakbari & Shahidi, 2008). Briefly, 0.5 mg of β‐carotene was dissolved in 1 ml of chloroform, 25 μl of linoleic acid, and 200 mg Tween 40. The chloroform was completely evaporated; then, 100 ml of aerated distilled water was added and the mixture was shaken. The sample (2 g/L) was dissolved in DMSO, and 350 µl of sample solution was added to 2.5 ml of the resulted mixture and then incubated in a water bath at 50°C for 2 hr with blanks. BHT was used as a positive control and DMSO as a negative control. The absorbance was measured at 470 nm, and the antioxidant activities (*I*%) were calculated using the following formula:

$$I\%=\frac{A_{\beta -\text{carotene}\;\text{after}\;2\;h\;\text{assay}}}{A_{\text{initial}\;\;\beta -\text{carotene}}}\times 100,$$

*A*_β‐carotene after 2 hr assay_: absorbance of β‐carotene after 2 hr assay; *A*_initial β‐Carotene_: absorbance of β‐carotene at the beginning of the experiments.

### 2.7 Statistical analysis

All experiments were repeated three times, and the results were analyzed by one‐way ANOVA test (*ρ* < .05) using STATISTICA version 6.

## 3 RESULTS AND DISCUSSION

### 3.1 Yield, chemical, physical, and organoleptic properties of *L. nobilis* EO

The yield of *L*. *nobilis* EO was calculated according to the plant dry matter. The yield was 1.13 ± 0.03% (Table 1). This value was comparable to that of 1.2% obtained by Haddouchi, Lazouni, Meziane, and Benmansour (2009) with *L*. *nobilis* from Tlemcen (Algeria) and to that of 1.1% on *L*. *nobilis* from Iran (Verdian‐rizi & Hadjiakhoondi, 2008). However, the yield of our EO was higher than that of 0.6% found in *L*. *nobilis* from El Kala, Algeria (Ouibrahim et al., 2013). On the contrary, the EO yield was lower than that given by the same species in different regions of Morocco, whose performance can reach 2.5% (Yilmaz, Timur, & Aslim, 2013). Vekiari et al. (2002) revealed that the harvest period, drying time, and extraction method are among other factors that may influence EO yields. Despite the importance of extraction yield, the commercial value of an EO is estimated in most cases by its organoleptic qualities (appearance, odor, and color) and biological activities, as well as by a number of constants called physical and chemical indices. These constants have been standardized by certain national and international organizations (AFNOR, ISO) and existing pharmacopoeias. The EO of *L*. *nobilis* presented a liquid, limpid, mobile and yellow appearance, and a typical aromatic odor. Physical and chemical criteria were determined, namely density, refractive index, acid index, and ester index (Table 1). These physical and chemical characteristics constitute a means of verification and quality control of the EO. Our tested *L*. *nobilis* EO showed a polyphenol value of 26.4 ± 0.22 μg/mg by the Folin–Ciocalteu colorimetric method.

**Table 1:** Yield, organoleptic, chemical, and physical properties of *Laurus nobilis* essential oil

Yield (% dry weight)	Organoleptic properties	Chemical and physical properties^a^, ^b^ (*M* ± *SD*)
1.13 ± 0.03	Odor: aromatic, pleasant Aspect: liquid, clear and mobile Color: transparent yellow	Density (20°C): 0.916 ± 0.006 Refractive index (20°C): 1.467 ± 0.002 Acid index: 5.1 ± 0.05 Ester index: 40.6 ± 0.35 Total phenols: 26.4 ± 0.22 μg/mg

^a^ AFNOR standard: AFNOR NF ISO 279:1999 (T75‐111).

^b^ AFNOR standard: AFNOR NF ISO 280:1999 (T75‐112).

The presence of phenolic compounds in botanical products such as EOs has been gaining much attention due to their antioxidant, antimicrobial, and flavoring properties in foods. Yilmaz et al. (2013) found that phenol contents in *L. nobilis* EO were 112.3 μg/mg, which are higher than that found in our study (26 μg/mg). The differences found in the phenol contents are not surprising, considering the different extractive methods that were applied. On the other hand, the influence of the plant material, in terms of genotype, geographic origin, and environmental conditions, cannot be excluded. Climatic factors influence certainly the phenol contents of EO. At industry level, it would be very important to ensure the biological efficiency of EOs based on their biophenols content.

### 3.2 **GC**–**MS analysis of *L*. *nobilis* EO**

Gas chromatography–mass spectrometry analysis (Table 2) showed that *L*. *nobilis* EO was characterized by 1,8‐cineole (35.5%), camphene (13.4%), linalool (11.4%), methyl eugenol (7.8%), sabinene (4.8%), α‐pinene (4.7%), *β*‐pinene (3.2%), and terpinen‐4‐ol (3.2%) as major constituents. Some reports were found on the composition of *L*. *nobilis* EO. Kilic, Kollmannsberger, and Nitz (2005) found that the main components in EO of *L*. *nobilis* of Turkey were 1,8‐cineole (24.2%–32.1%), sabinene (7.1%–7.6%), *α*‐terpinyl acetate (4.8%–6.5%), *α*‐pinene (3.9%–5.0%), and *β*‐pinene (3.0%–3.8%). The main compounds for *L*. *nobilis* EO from Tunisia were 1,8‐cineole (42.3%), *α*‐terpinyl acetate (11.2%), *α*‐pinene (7.8%), *β*‐pinene (5.9%), and sabinene (5.4%) (Bouzouita et al., 2001). Again in Tunisia, Snuossi et al. (2016) reported that the main constituents of *L*. *nobilis* EO were 1,8‐cineole (56%), *α*‐terpinyl acetate (9.0%), 4‐terpineol (5.2%), methyl eugenol (3.6%), sabinene (3.5%), and *α*‐pinene (3.2%). Italian *L*. *nobilis* EO was mainly composed of 1,8‐cineole (35.7%), *E*‐sabinene hydrate (9.7%), *α*‐terpinyl acetate (9.3%), methyl eugenol (6.8%), and sabinene (6.5%) (Flamini et al., 2007). Caputo et al. (2017) found that 1,8‐cineole (31.9%), sabinene (12.2%), and linalool (10.2%) were the main components in Italian *L*. *nobilis* EO. The composition of EOs from any species varies according to genotypes, environmental factors, age of plant, and time of harvesting (Prakash et al., 2012). Such a variation in chemical composition of EOs would definitely alter their biological activity Therefore, in utilizing EOs for the preservation of food commodities, it is necessary to characterize the chemical composition for the standardization of the product.

**Table 2:** Chemical composition of the essential oil from *Laurus nobilis*

Components	Retention indices	Percentage (%)	Formula
*α*‐Phellandrene	920	0.6	C_10_H_16_
*α*‐Pinene	928	4.7	C_10_H_16_
Camphene	945	13.4	C_10_H_16_
Sabinene	967	4.8	C_10_H_16_
*β*‐Pinene	973	3.2	C_10_H_16_
Myrcene	983	0.2	C_10_H_16_
*δ*‐3‐Carene	1,005	0.6	C_10_H_16_
*α*‐Terpinolene	1,013	0.5	C_10_H_16_
*p*‐Cymene	1,015	0.2	C_10_H_14_
m‐Cymene	1,021	0.8	C_10_H_14_
Limonene	1,024	1.3	C_10_H_16_
1,8‐Cineole	1,028	35.5	C_10_H_18_O
*γ*‐Terpinene	1,052	0.9	C_10_H_16_
*trans*‐Sabinene hydrate	1,066	0.3	C_10_H_18_O
Linalool	1,098	11.4	C_10_H_18_O
*δ*‐terpineol	1,169	0.2	C_10_H_18_O
Borneol	1,171	0.4	C_10_H_18_O
Terpinen‐4‐ol	1,178	3.2	C_10_H_18_O
*α*‐Terpineol	1,194	2.2	C_10_H_18_O
Nerol	1,223	0.2	C_10_H_18_O
Zeta‐Fenchene	1,243	0.2	C_10_H_16_
Eugenol	1,350	1.0	C_10_H_12_O_2_
*β*‐Elemene	1,383	0.3	C_15_H_24_
Methyl Eugenol	1,398	7.8	C_11_H_14_O_2_
*β*‐Caryophyllene	1,412	0.6	C_15_H_24_
Cinnamic acid	1,466	0.3	C_9_H_8_O_2_
Bicyclogermacrene	1,489	0.2	C_15_H_24_
*δ*‐Cadinene	1,513	0.2	C_15_H_24_
12‐Nor‐caryophyll‐5‐en‐2‐one	1,531	0.2	C_14_H_22_O
Elemicin	1,545	0.6	C_12_H_16_O_3_
Spathulenol	1,574	0.9	C_12_H_24_O
Caryophyllene oxide	1,576	0.6	C_15_H_24_
Valencene	1,591	0.2	C_15_H_24_
(E,Z)‐1,2‐diethylidenecyclopentane	1,600	0.2	C_9_H_14_
*t*‐Muurolol	1,655	0.7	C_15_H_25_O
Longifolene	1,693	0.2	C_15_H_24_
Total identified (%)		98.8	
Monoterpene hydrocarbons (%)		31.6	
Oxygen‐containing monoterpenes (%)		64.2	
Sesquiterpene hydrocarbons (%)		2.3	
Oxygen‐containing sesquiterpenes (%)		0.7	

### 3.3 Antifungal activity of the EO from *L*. *nobilis*

**Figure 2:**
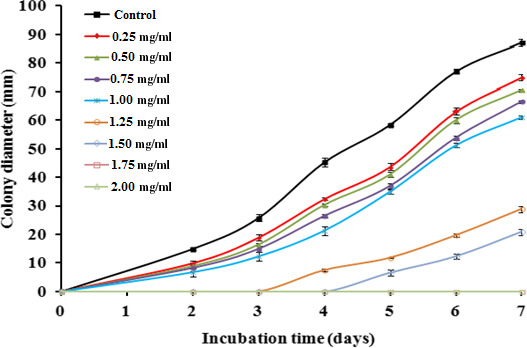
Influence of different concentrations of *Laurus nobilis* essential oil on *Aspergillus flavus* growth during 7 days of incubation

**Figure 3:**
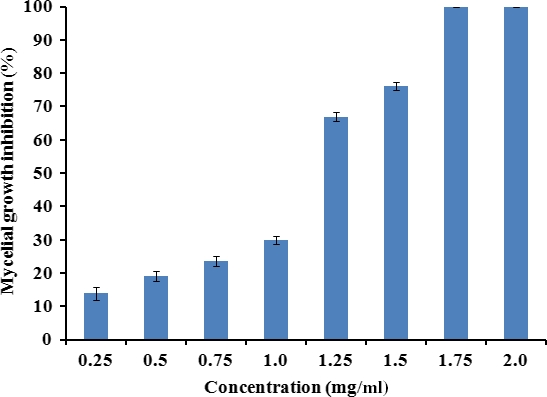
Percentage inhibition of *Aspergillus flavus* growth after 7 days of incubation

The antifungal activity of *L*. *nobilis* EO against *A*. *flavus* during the seven days of incubation is shown in Figure 2. As compared to the control, the colony diameter was significantly reduced (*p* < .05) with increasing concentrations of EO from 0.25 to 1.5 mg/ml in a dose‐dependent manner. Also, the onset of fungal growth was delayed 3 days at 1.25 mg/ml and 4 days at 1.5 mg/ml, while the fungus growth was totally inhibited with the higher treatments of 1.75 and 2 mg/ml. The percentage (%) inhibition of mycelia growth after day 7 of incubation is presented in Figure 3. The analysis of data revealed that *L*. *nobilis* EO at 1.5 mg/ml caused 76.1% inhibition in mycelial growth of *A*. *flavus* as compared with the control (*p* < .05), while the inhibition reached 100% at concentrations of 1.75 mg/ml and above.

The MIC and MFC techniques were employed to assess fungistatic and fungicidal properties of the EO against a series of foodborne fungi. *L*. *nobilis* EO was tested up to a maximum concentration of 2 mg/ml. The data of fungitoxic spectrum are shown in Table 3. The MIC against the toxigenic strain of *A*. *flavus* was recorded at 1.75 mg/ml, while MFC attained 2 mg/ml. The MIC of the EO against the other fungal species ranged between 1.00 and 1.91 mg/ml, while the MFC varied from 1.25 to >2 mg/ml. Aligiannis, Kalpoutzakis, Mitaku, and Chinou (2001) proposed classification for plant materials, based on MIC results as follows: (a) strong inhibitors MIC up to 0.5 mg/ml, (b) moderate inhibitors MIC between 0.6 and 1.5 mg/ml, and (c) weak inhibitors MIC above 1.6 mg/ml.

**Table 3:** Antifungal activity of the essential oil from *Laurus nobilis*

Fungal species	MIC (mg/ml)	MFC (mg/ml)
*Aspergillus flavus*	1.75 ± 0.19	2.00 ± 0.00
*Aspergillus carbonarius*	1.58 ± 0.14	1.58 ± 0.38
*Aspergillus fumigatus*	1.00 ± 0.25	1.41 ± 0.14
*Aspergillus niger*	1.50 ± 0.25	>2.00
*Aspergillus ochraceus*	1.33 ± 0.14	1.66 ± 0.28
*Aspergillus tamarii*	1.25 ± 0.00	1.25 ± 0.25
*Aspergillus terreus*	1.16 ± 0.28	1.25 ± 0.50
*Fusarium* sp.	1.91 ± 0.14	>2.00
*Penicillium* sp.	1.08 ± 0.14	1.25 ± 0.25
*Rhizopus* sp.	1.58 ± 0.38	>2.00

Note

Values are *M* ± *SD* representative of three independent experiments.

Abbreviations: MFC, minimum fungicidal concentration; MIC, minimum inhibitory concentration.

Ben Miri, Belasli, Djenane, and Ariño (2019) recently reviewed the antifungal effect of some EOs on the growth of *A. flavus*. The antifungal activity of our *L. nobilis* EO is comparable to that of callistemon, cardamom, lemongrass, and verbenacea, which are in the range of 0.25 to 1 mg/ml. In present investigation, *L*. *nobilis* EO showed moderate antifungal activity and corroborated other works on the antifungal potential of this EO, such as that by Guynot, MarÍn, SetÚ, Sanchis, and Ramos (2005) who obtained similar antifungal activity of *L*. *nobilis* EO against the growth of *Aspergillus* sp. In contrast, weaker antimicrobial effects were reported by Tajkarimi, Ibrahim, and Cliver (2010). Likewise, Simić et al. (2004) tested the antifungal potential of the *Aniba rosaeodora* and *L. nobilis* EOs against fungal species. The EO of *A*. *rosaeodora* showed higher antifungal effects than that of *L*. *nobilis*. According to these authors, the difference resides in that linalool was the main component in the EO of *A*. *rosaeodora*, while 1.8‐cineole was dominant in *L. nobilis*. In this sense, GC‐MS analysis showed that tested *L*. *nobilis* EO was characterized by 1,8‐cineole as major compound (Table 2). Hmiri et al. (2011) reported that the antifungal activity of *E*. *camaldulensis* rich in 1,8‐cineole was due at least partially to the action of this monoterpene, and the works of Shukla, Singh, Prakash, and Dubey (2012) and Caputo et al. (2017) showed that pure 1,8‐cineole inhibited mycelial growth but at higher concentrations than complete EOs. According to Gusarov, Shatalin, Starodubtseva, and Nudler (2009), 1,8‐cineole causes hyphal morphological changes and damage to the plasma membrane by permeabilizing the membrane. Nevertheless, the antifungal activity of our EO could have been synergistically contributed by the presence of monoterpene alcohols such as linalool and terpineol. Several authors reported that these compounds increase the permeability of the plasma membrane and inhibit the respiration process on the mitochondrial membrane of fungi (Deba, Xuan, Yasuda, & Tawata, 2008; Imelouane et al., 2009).

The study of MIC and MFC is important to determine the minimum dose to control fungal populations and gives opportunity for EOs to come in close contact with fungal spores in the medium (Kumar, Shukla, Singh, Prasad, & Dubey, 2008). The antifungal activity of EOs can be adequately evaluated by the broth dilution method, though this method requires a large amount of EO (Mesa‐Arango et al., 2009). Da Silva Bomfim et al. (2020) found that *Rosmarinus officinalis* EO exhibited antifungal activity against *A*. *flavus* with both MIC and MFC values around 0.5 mg/ml, in a similar range of MIC and MFC values found in our study.

In general, it is difficult to correlate the antifungal activity with single compounds or classes of compounds. EOs consists of a wide variety of chemical compounds, making it difficult to establish a relationship between a specific compound and a single target in the cell. There is evidence that even minor components play a critical role in antimicrobial activities, and it appears that the inhibitory effects are the result of their synergistic action (Cabral et al., 2013; Djenane et al., 2019). Technological advances have been recorded in recent years in terms of the fight and prevention against toxigenic molds. In this context, Kumar, Kujur, Singh, and Prakash (2019) observed that the encapsulation of a mixture of different EO components including thymol, linalool, and methyl cinnamate increased the antifungal activity against several foodborne molds. Peixoto et al. (2017) demonstrated the antifungal potential of the chemically characterized EO of *L. nobilis* L. against *Candida* spp. biofilm adhesion and formation, due to the presence of monoterpenes and sesquiterpenes in its composition.

### 3.4 Antiaflatoxin activity of the EO from *L*. *nobilis*

**Figure 4:**
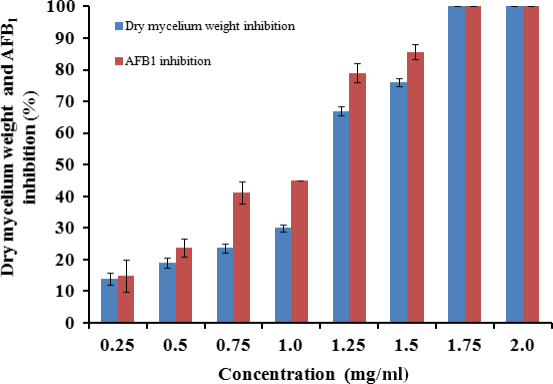
Effect of *Laurus nobilis* essential oil on dry mycelium weight and aflatoxin B1 (AFB_1_) production by *Aspergillus flavus* during 10 days of incubation

The mycelium dry weight and the production of AFB_1_ were measured after an incubation period of 10 days, as shown in Figure 4. The recorded data explain an inversely proportional relationship between the amount of EO and the mycelium dry weight the AFB_1_ production. As the dose of EO increased in the SMKY broth, the inhibition of mycelium dry weight was accompanied by a decrease in the synthesis of AFB_1_. The data revealed that both mycelium dry weight and AFB_1_ production were significantly inhibited (*p* < .05) by EO concentrations between 0.25 and 1.50 mg/ml as compared to the control. The percentage of inhibition ranged from 11.1% to 80.9% for mycelium dry weight and from 14.9% to 85.7% for AFB_1_ production. A complete growth inhibition was observed at EO concentrations above 1.75 mg/ml, so no aflatoxin was detected.

A positive correlation was found between the decrease in mycelium dry weight and the inhibition of AFB_1_ with increasing concentrations of *L*. *nobilis* EO. This may be due to the limiting effect of different bioactive EO constituents on vegetative cells and spores during fungal development, as suggested by Tzortzakis and Economakis (2007). Similarly, Da Silva Bomfim et al. (2020) found that *Rosmarinus officinalis* EO (0.5 mg/ml) was effective in the reduction of mycelial growth and the production of ergosterol, as well as in the inhibition of the production of aflatoxins by *A*. *flavus*.

The low molecular weight and highly lipophilic compounds of EOs pass easily through cell membranes and disrupt the fungal cell organization (Shukla et al., 2012). The aflatoxin IC of the EO was found to be the same as the IC required for the reduction in mycelial dry weight supporting the findings of some workers (Mishra et al., 2013; Reddy, Reddy, & Muralidharan, 2009). The inhibition mechanism of AFB_1_ production is not very clear. *L*. *nobilis* EO may interfere with some steps in the metabolic pathways of the *A*. *flavus*, which controls AFB_1_ biosynthesis. Oliveira, Carvajal‐Moreno, Correa, and Rojo‐Callejas (2020) found that thyme EO could inhibit growth and AFB_1_ production in *A*. *flavus* by gene suppression of fungal secondary metabolism, causing cell death by apoptotic mechanisms. In addition, Nazareth et al. (2020) and Wang et al. (2019) confirmed that certain plant biomolecules alter the gene transcriptomes involved in the synthesis of AFB_1_. It has been shown that the AFB_1_ biosynthesis genes are the possible site of molecular action of the bioactive molecules contained in EOs (Kujur, Yadav, Kumar, Singh, & Prakash, 2019; Yuan et al., 2019). On the other hand, the EO of *Pimenta dioica* caused reduction of methylglyoxal (an AFB_1_ inducer) and enhanced membrane ions leakage (Ca^2+^ K^+^ and Mg^2+^), as reported by Chaudhari et al. (2020). The oil exhibited complete protection of stored maize from fungal infestation (without affecting seed germination) and subsequent AFB_1_ production at 2.5 and 1.5 µl/ml, respectively.

### 3.5 Application of *L*. *nobilis* EO in stored wheat grains: Protection against *A*. *flavus* by fumigation

**Figure 5:**
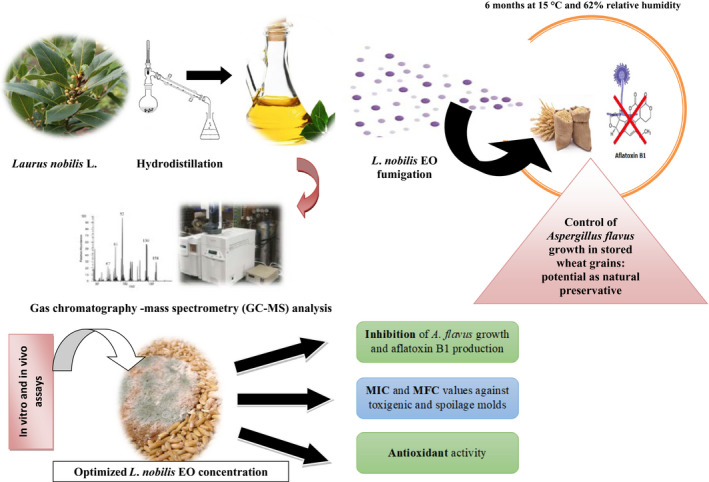
Experimental design: Optimized essential oil (EO) dose (extracted from Algerian *Laurus nobilis*) application in stored wheat grains as antifungal and aflatoxin production purposes

The experimental design is shown in Figure 5. As described in Section 2.5, the EO was obtained from Algerian *L*. *nobilis*, and lots of wheat grains samples were fumigated with this EO as potential natural preservative. Wheat grains were inoculated with pathogenic fungi (*A*. *flavus*) and stored during 6 months at 15°C and 62% relative humidity. Wheat grains samples were analyzed for *A*. *flavus* growth. The potential use of *L*. *nobilis* EO as wheat preservative was based on the percentage of protection against *A*. *flavus* in *L*. *nobilis* EO‐fumigated wheat grains as compared to untreated samples (control).

The EO showed remarkable efficacy in fumigated wheat samples during storage for up to 6 months providing from 51.5% to 76.7% protection of wheat grains from *A*. *flavus* contamination (Table 4). In general, to obtain similar protective effects in food products as those observed in vitro, higher concentrations of EOs should be used (Kedia et al., 2014; Tian et al., 2011). This can be explained by the fact that when the EOs are in contact with the food matrix, some active volatile substances are bound by food components. In addition, food lipids can form a coating around the microorganisms, protecting them from antimicrobial agents. Also, the lower water content of grains as compared to laboratory media could hinder the transfer of antimicrobial molecules to the active site in the microbial cell (Prakash, Mishra, Kedia, & Dubey, 2014). Thus, it has been hypothesized that penetration of oils into the internal parts of the grain could improve in the presence of water, and thus, toxigenic microorganisms could be more easily controlled in the inner parts of moist seeds. Therefore, the nature of the food system, the density of the fungal inoculum, the storage conditions, and their moisture content must be taken into consideration when determining the in vivo concentration of EOs against food contamination by molds.

**Table 4:** Percent (%) protection of wheat grains fumigated with *Laurus nobilis* essential oil (EO) after 6 months of storage

Wheat seed samples	*Aspergillus flavus* (cfu/g × 10^3^)		Protection (%)
	Control	EO‐fumigated	
HD1220 (Hiddab)	43	10	76.7
AS 81,189 A (Ain Abid)	33	16	51.5

It seems that EOs with high levels of phenolic compounds have more important antifungal activities. According to Prakash et al. (2012), EOs should be more beneficial than the synthetic preservatives because of their biodegradable nature. EOs are a mixture of different major and minor components that act together for their biological activities. For this reason, there is less chance of developing resistant fungal strains as reported for many synthetic preservatives. Currently, many formulations of branded EOs could thus be used in agricultural crops according to the specifications of organic farming. EOs are volatile, so vapors can easily be removed from fumigated foods after drying in the sun. In this regard, active packaging and microencapsulation technologies can be used with EOs for the storage of foodstuffs (Bouzidi, Lakhlef, Hellal, & Djenane, 2019; Djenane et al., 2019; García‐Díaz, Patiño, Vázquez, & Gil‐Serna, 2019; Li et al., 2019).

In conclusion, the EO of laurel leaves could play an important role in stored wheat grains protection, reducing risks associated with the use of synthetic insecticides. Given the presumed negative impact of synthetic fungicides on human health, the use of plant‐based antifungal agents has generated considerable interest in the agri‐food industries.

### 3.6 Antioxidant activity of the EO from *L*. *nobilis*

Essential oils from laurel leaves have been widely studied for their antioxidant activity. Moreover, some authors have used different extractive and laboratory methods in order to enhance the antioxidant activities. *L*. *nobilis* EO showed free radical scavenging activity in dose‐dependent manner, and its IC_50_ value was 602 ± 5.8 μg/ml. For comparison, the IC_50_ value of synthetic antioxidant BHT used as positive control was 306 ± 4.5 μg/ml. Snuossi et al. (2016) reported that EO from *L*. *nobilis* harvested in Tunisia had an effective antioxidant activity at 135 μg/ml. Similarly, Boughendjioua (2017) reported that Algerian *L*. *nobilis* EO possessed effective DPPH scavenging activity at the level of 94.93 μg/ml, whereas Cherrat et al. (2014) found that Moroccan *L*. *nobilis* EO possessed effective DPPH scavenging activity at low concentrations of 1.25 to 10 μg/ml. According to Yilmaz et al. (2013), Turkish *L*. *nobilis* EO gave an IC_50_ of 59.2 μg/ml. These differences in IC_50_ can be attributed to analytical methodologies and to the several factors influencing the chemical composition of EO such as the variety, plant growth conditions, EO storage conditions, and the extraction methods used.

Numerous studies have shown that the biological activities of EOs of aromatic plants are related to their chemical composition and in particular to the major compounds (Yadav et al., 2019). However, minority compounds can interact directly, or in a synergistic or antagonistic manner, to create a mixture with biological activity. Several compounds that we have detected in *L*. *nobilis* EO have already been described as antioxidants. These include linalool and 1,8‐cineole (Wang, Chen, Sciarappa, Wang, & Camp, 2008), terpene‐4‐ol, *α*‐pinene, and *β*‐pinene (Singh, Batish, Kaur, Arora, & Kohli, 2006). Durazzo and Lucarini (2018) indicated that antioxidant properties are an expression of the interactions between bioactive molecules and other components of a food matrix. The interactions can give a combined and synergistic effect, an antagonistic effect, or no additional effect. Applied to EOs, the state of the art for investigation of antioxidant properties include the identification, isolation, and quantification of biologically active compounds present in food matrices as starting point, and this is followed by assessment of their interactions. This study approach will be especially important regarding the possible development of a natural EO as plant‐based food biopreservative.

In the β‐carotene bleaching assay, the oxidation of linoleic acid was moderately inhibited by the EO (46 ± 1.4%) as compared to positive control BHT (95 ± 1.6%). Data of β‐carotene bleaching assay were around 15% lower than that provided by the radical scavenging activity.

When exposed to environmental oxidative stress, molds activate several lines of defense to protect against the cellular damage that reactive oxygen species (ROS) can cause. It has been suggested that the first line of defense is represented by the activation of antioxidant enzymes such as catalase (CAT), superoxide dismutase (SOD), and peroxidase. Modulation of CAT and SOD activity was directly associated with a change in ROS levels but also with a modulation of aflatoxin production in *A*. *flavus* (Narasaiah, Sashidhar, & Subramanyam, 2006). In addition, it has been shown that aflatoxin production is induced by an increase in oxidative stress (ROS) in *A*. *flavus* and *A*. *parasiticus* and that aflatoxinogenic strains require higher levels of oxygen compared to nonaflatoxinogenic strains (Jayashree & Subramanyam, 2000).

Lipid peroxidation is a consequence of ROS formation, and it has been shown to be involved in the synthesis of aflatoxin. Kim et al. (2008) showed that the biosynthesis of aflatoxins is related to the formation of ROS and the peroxidation of fungal cells. On the other hand, Ferreira et al. (2013) reported that the mechanism of inhibition of Curcuma longa EO and curcumin on the production of aflatoxins might be related to the inhibition of the ternary steps of aflatoxin biosynthesis involving the peroxidation and oxygenation of lipids. This could therefore have contributed to the observed antiaflatoxinogenic effect of the EO from *L. nobilis*. Our results showed significant antioxidant activity, so the high AFB_1_ inhibitory efficacy may be partly due to its antioxidant properties on lipid peroxidation in the biosynthesis process of AFB_1_.

## 4 CONCLUSION

Essential oils are increasingly used in the food industry as an alternative of synthetic products, as natural food preservatives to extend product shelf life since they have antibacterial, antifungal, and antioxidant properties. The safety risk in stored grains, such as the presence of toxigenic molds and mycotoxins, is a major concern for food safety. The frequency of contamination of stored wheat by aflatoxins shows that current control strategies are insufficient to guarantee food safety and that it is necessary to develop other complementary agri‐food technologies that are more sustainable than currently implemented ones. In this context, a natural alternative strategy was developed to prevent the growth of toxigenic molds during the storage of wheat grains. *L*. *nobilis* EO led to noticeably inhibition of *A*. *flavus* and AFB_1_ production in vitro, as well as suppression of common foodborne fungal species. In stored wheat grains, the fumigation with *L*. *nobilis* EO reduced *A*. *flavus* counts in the treated grains. In addition, the EO showed high antioxidant activity. In this respect, the use of biopreservation to extend shelf life during wheat storage could be a sustainable solution for the development of Algerian Agri‐food industries to reduce losses of products due to fungal growth and mycotoxin contamination.

The conclusions of this study justify future research for the application of EOs as fumigants in food systems to improve their safety and shelf life by controlling spoilage and toxigenic molds as well as lipid peroxidation. These findings should be taken into account in the development of safe and eco‐friendly specific technologies such as new biopolymers and nanoparticles coatings.

## CONFLICT OF INTEREST

The authors declare that they have no competing interests.

## AUTHORS’ CONTRIBUTIONS

AB, YBM, MA, and LAO were involved in experimental and analytical work. LM, DD, and AA were involved in the designing of the experiment, data analysis, and interpretation of the results. All authors reviewed and approved the final manuscript.

## ETHICAL APPROVAL

The experiment does not include any animal or human testing.
